# Antibiotics and activity spaces: protocol of an exploratory study of behaviour, marginalisation and knowledge diffusion

**DOI:** 10.1136/bmjgh-2017-000621

**Published:** 2018-03-28

**Authors:** Marco J Haenssgen, Nutcha Charoenboon, Giacomo Zanello, Mayfong Mayxay, Felix Reed-Tsochas, Caroline O H Jones, Romyen Kosaikanont, Pollavat Praphattong, Pathompong Manohan, Yoel Lubell, Paul N Newton, Sommay Keomany, Heiman F L Wertheim, Jeffrey Lienert, Thipphaphone Xayavong, Penporn Warapikuptanun, Yuzana Khine Zaw, Patchapoom U-Thong, Patipat Benjaroon, Narinnira Sangkham, Kanokporn Wibunjak, Poowadon Chai-In, Sirirat Chailert, Patthanan Thavethanutthanawin, Krittanon Promsutt, Amphayvone Thepkhamkong, Nicksan Sithongdeng, Maipheth Keovilayvanh, Nid Khamsoukthavong, Phaengnitta Phanthasomchit, Chanthasone Phanthavong, Somsanith Boualaiseng, Souksakhone Vongsavang, Rachel C Greer, Thomas Althaus, Supalert Nedsuwan, Daranee Intralawan, Tri Wangrangsimakul, Direk Limmathurotsakul, Proochista Ariana

**Affiliations:** 1 Centre for Tropical Medicine and Global Health, Nuffield Department of Clinical Medicine, University of Oxford, Oxford, UK; 2 CABDyN Complexity Centre, Saïd Business School, University of Oxford, Oxford, UK; 3 Green Templeton College, University of Oxford, Oxford, UK; 4 Mahidol Oxford Tropical Medicine Research Unit, Faculty of Tropical Medicine, Mahidol University, Bangkok, Thailand; 5 School of Agriculture, Policy and Development, University of Reading, Reading, UK; 6 Lao Oxford Mahosot Wellcome Trust Research Unit (LOMWRU), Mahidol Oxford Tropical Medicine Research Unit (MORU), Faculty of Tropical Medicine, Mahidol University, Bangkok, Thailand; 7 Faculty of Postgraduate Studies, University of Health Sciences, Vientiane, Laos; 8 Institute for New Economic Thinking, Oxford Martin School, University of Oxford, Oxford, UK; 9 Department of Sociology, University of Oxford, Oxford, UK; 10 Department of Health System and Research Ethics, KEMRI Wellcome Trust Research Programme, Kilifi, Kenya; 11 School of Social Innovation, Mae Fah Luang University, Chiang Rai, Thailand; 12 School of Liberal Arts, Mae Fah Luang University, Chiang Rai, Thailand; 13 Salavan Provincial Hospital, Salavan, Laos; 14 Oxford University Clinical Research Unit (OUCRU), Ho Chi Minh City, Vietnam; 15 Medical Microbiology Department, Radboudumc, Nijmegen, The Netherlands; 16 National Human Genome Research Institute, National Institutes of Health, Bethesda, Maryland, USA; 17 Department of Global Health and Development, London School of Hygiene & Tropical Medicine, London, UK; 18 Primary Care Department, Chiangrai Prachanukroh Hospital, Chiang Rai, Thailand

**Keywords:** antimicrobial resistance, treatment-seeking behaviour, marginalisation, activity space, social research, survey, qualitative research, Thailand, Lao PDR

## Abstract

**Background:**

Antimicrobial resistance (AMR) is a global health priority. Leading UK and global strategy papers to fight AMR recognise its social and behavioural dimensions, but current policy responses to improve the popular use of antimicrobials (eg, antibiotics) are limited to education and awareness-raising campaigns. In response to conceptual, methodological and empirical weaknesses of this approach, we study people’s antibiotic-related health behaviour through three research questions.

RQ1: What are the manifestations and determinants of problematic antibiotic use in patients’ healthcare-seeking pathways?

RQ2: Will people’s exposure to antibiotic awareness activities entail changed behaviours that diffuse or dissipate within a network of competing healthcare practices?

RQ3: Which proxy indicators facilitate the detection of problematic antibiotic behaviours across and within communities?

**Methods:**

We apply an interdisciplinary analytical framework that draws on the public health, medical anthropology, sociology and development economics literature. Our research involves social surveys of treatment-seeking behaviour among rural dwellers in northern Thailand (Chiang Rai) and southern Lao PDR (Salavan). We sample approximately 4800 adults to produce district-level representative and social network data. Additional 60 cognitive interviews facilitate survey instrument development and data interpretation. Our survey data analysis techniques include event sequence analysis (RQ1), multilevel regression (RQ1–3), social network analysis (RQ2) and latent class analysis (RQ3).

**Discussion:**

Social research in AMR is nascent, but our unprecedentedly detailed data on microlevel treatment-seeking behaviour can contribute an understanding of behaviour beyond awareness and free choice, highlighting, for example, decision-making constraints, problems of marginalisation and lacking access to healthcare and competing ideas about desirable behaviour.

**Trial registration number:**

NCT03241316; Pre-results.

**Summary box**

- Antimicrobial resistance (AMR) is a global health priority, and leading UK and global strategy papers recognise its social and behavioural dimensions.
- Behavioural elements of these strategy papers have conceptual, methodological and empirical weaknesses.
- We will carry out social research to understand the nature of antibiotic-related treatment-seeking behaviour in rural Thailand and Lao PDR.
- We will conduct survey research with 4800 adult villagers (yielding district-level representative and social network data), complemented with cognitive interviews and secondary administrative data.
- Our study will contribute to the nascent yet urgently needed social research in AMR.

## Background

Access to non-prescription antibiotics is a widespread phenomenon in low-income and middle-income countries (LMICs),^1^ contributing to inappropriate medicine use, to the development of antimicrobial resistance (AMR) and potentially to the subsequent spread of resistant bacteria across the world.^2^ Leading global and UK policy papers aiming to deal with the overuse and misuse of antibiotics among the general population focus thereby wholly on educational and awareness-raising campaigns to encourage positive behaviour change.^3–6^ Awareness raising is important,^7^ but as the sole global strategy focusing on people’s healthcare-seeking behaviour (aside from public health interventions to prevent illness), it has three central weaknesses.

The first is conceptual: by limiting our attention to ‘awareness’ as the main driver of people’s antibiotic use, we are prone to neglecting determinants of health behaviour beyond information and free choice such as economic constraint, social pressure or local conceptions of illness.^8–11^ However, little is known about how economic constraints, social discrimination or spatial marginalisation deprive people of choices and drive them into seemingly adverse antibiotic-related behaviours, and whether and how interventions should address these constraints in the context of global AMR policy.

The second is methodological: quantitative community-level and population-level analyses of antibiotic usage disregard routinely that healthcare processes involve combinations of ‘no care’, ‘self-care’ and healthcare from many different practitioners.^12^ Although conceptually established and applied in qualitative research,^13 14^ the sequential understanding of treatment-seeking behaviour has not yet entered quantitative public health research. The majority of quantitative analyses of healthcare behaviour in LMICs instead adopt a single-stage approach, implying that a patient ‘chooses’ once from a portfolio of healthcare options, some of which may be more likely to involve antibiotics than others.^15 16^ This conventional analysis can be useful to measure rates of antibiotic access, but their aggregate nature forgoes valuable information and obscures the factors influencing antibiotic use throughout an illness, for example, the use of information technology to gather information (as we demonstrate in Haenssgen and Ariana^17^).

The third is empirical: studies of awareness campaign effectiveness focus on knowledge gains but disregard the social mechanisms of information diffusion (see, eg, Onnela *et al*^18^). Awareness campaigns often expect information to spread within communities, but these communities are not always collaborative.^19^ In addition, as a potential solution in a healthcare-seeking problem, new information about antibiotic use also competes with other ideas, some of which may represent dominant healthcare strategies from the individual’s perspective.^20^^i^ We do not understand sufficiently how these interdependencies play out during the diffusion process of antibiotic knowledge and practice. It therefore appears risky to confine our behavioural tactics to the single mechanism of awareness raising that is merely *believed* to function.

In response to these conceptual, methodological and empirical challenges, we intend to improve the understanding of patients’ antibiotic-related behaviour to support creative thinking about targeted and unconventional AMR interventions in LMICs. Three research questions will guide our enquiry.

RQ1: What are the manifestations and determinants of problematic antibiotic use in patients’ healthcare-seeking pathways?

RQ2: Will people’s exposure to antibiotic awareness activities entail changed behaviours that diffuse or dissipate within a network of competing healthcare practices?

RQ3: Which proxy indicators facilitate the detection of problematic antibiotic behaviours across and within communities?

We adhere to conventions in the field of public health when using the contentious language of ‘problematic’ and ‘appropriate’ behaviour. However, from a behavioural perspective, a conventional clinical definition of ‘problematic behaviour’ is impractical to pursue because it would involve claims on the mis/match between a patient’s condition (eg, being caused by a particular microorganism) and the type, dosage, duration and affordability of the administered drugs. Patients are not necessarily able to diagnose themselves, decide whether an antibiotic is needed and then select the clinically most suitable course of treatment. Indeed, many illnesses do not involve a doctor at all. Considering that ‘problematic behaviour’ is subjective and context specific, we instead record patients’ behavioural trajectories during an illness and apply different evaluative criteria to make judgements of ‘appropriateness’ flexibly and transparently. In consultation with the social anthropologists, medical practitioners and local field staff in our study team, we will categorise individually as well as socially ‘appropriate’ behavioural sequences that go beyond binary assessments of healthcare access or antibiotic use. For example, individually rational bypassing of referral systems could entail healthcare resource misallocation from a public health perspective, and individual antibiotic use can entail negative externalities on the societal level through potential contributions to antibiotic resistance. Our interest in human behaviour thereby does not intend to attribute blame to patients for patterns of antibiotic usage that might contribute to AMR, but rather to explore decisions and decision-making constraints on the healthcare demand side. The provision of the raw behavioural sequences will allow other researchers to make their own evaluations of health behaviours depending on their specific assessment criteria and interests.

## Methods

### Theoretical framework

Our study departs from conventional policy assumptions that antibiotic misuse among patients stems from a lack of knowledge regarding appropriate medicine use. Instead, we frame antibiotic use as one among multiple solutions in people’s healthcare ‘activity space’.

Contrary to existing applications of activity space frameworks in areas like disability and mobility^21–23^ and social geography,^24–26^ we do not adhere to spatial conceptualisations through which, for example, experienced space is linked to health outcomes like obesity or HIV.^24 26^ Instead, we draw on theoretical strands and techniques from the disciplines of public health, medical anthropology, sociology and development economics, which suggest that healthcare behaviour takes place within a physical and social space populated by various healthcare providers (including drug vendors), and that this space is defined by the difficulty and the perceived and dynamically changing value of utilising any of these providers during an illness. Difficulty is determined by the tools and solutions at the patients’ disposal (eg, social support networks, cars, communication technology), but not every solution affects access to different providers equally. In addition, patients might not be aware of every provider in their vicinity (they therefore do not enter the activity space), and some providers signal better healthcare value than others, depending on type and severity of the illness. Moreover, the activity space overlaps across patients and it is thus a shared space. These characteristics lead us to identify three key elements of antibiotic usage in a healthcare activity space: (1) the emergence of pathways through the health system during the course of an illness; (2) the coexistence of multiple solutions for the health problem, the value of which changes dynamically and (3) cooperation, competition and exclusion in a shared social space. As a result, our definition of activity spaces can be likened to ‘markets’ in the strategic marketing literature,^27^ where markets can be delineated by different customer groups (in the case of health, eg, different socioeconomic groups), the customer function to be served (eg, curative care) and the ‘alternative solutions’ available to fulfil this function (eg, antibiotic use at home, care from primary health centre, sick leave).

The breadth of the activity space framework allows us to consider multiple, and otherwise conceptually more restricted, explanatory approaches for treatment-seeking behaviour side-by-side (eg, transaction cost approaches alongside the information deficit arguments that often underlie policy narratives).^28 29^ The conceptualisation as a shared social space also permits us to go beyond conventional individualistic treatment-seeking models in order to explore new forms of health-related collective action problems. In addition, our framework permits us to examine the determinants of problematic behaviour and the positive as well as negative outcomes of technology use, rather than merely articulating the enabling conditions of desired behaviour change as is common in the public health literature.^30–32^ Activity spaces are therefore not a theory per se, but a useful analytical domain to guide our research.

### Research design

We will carry out population-level and community-level health behaviour surveys in rural Chiang Rai (Thailand) and Salavan (Lao PDR) and we collect complementary qualitative data. This will result in two survey data sets: the first contains district-level representative treatment-seeking behaviour of approximately 1200 adults across 30 rural communities per country; the second comprises social network censuses of approximately 400 adults each in three rural communities per country. Within the sampled villages, we will complete checklists about existing formal and informal healthcare facilities and gather patient load data from primary care units catering to the respective villages. As part of the questionnaire testing process, we will conduct (and collect as primary data) cognitive interviews to improve the survey tool and to interpret our data.

**Figure 1:**
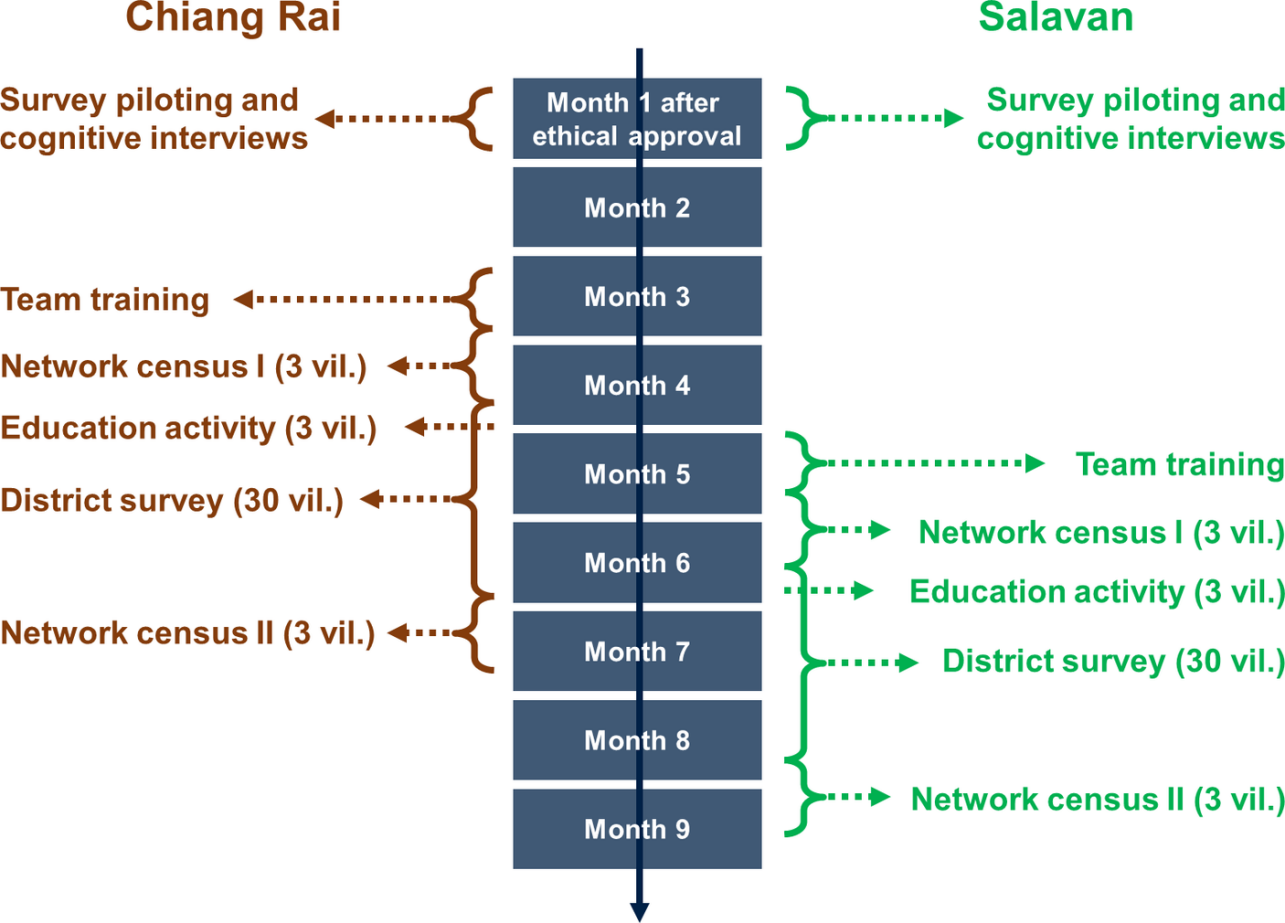
Study design and timeline.

We will carry out the district-level village survey in one round and the village-level social network censuses in two rounds (see figure 1; source: authors). Between the two village social network censuses, we will carry out education activities in the selected villages as part of antibiotic-related and medicine-use-related public engagement. Developed after a year of qualitative health behaviour research across Southeast Asia, these small-scale activities aim to help villagers learn more about drug resistance and to help the social and medical research communities to better appreciate local people’s access to healthcare and medicine conceptions and constraints. The activities will take place after the network surveys in each of the network villages, lasting 1–2 days, involving approximately 30 villagers each, and including interactive sessions like trading games, poster making, storytelling, and role plays.^ii^ The district survey will take place after the education activities, and we will subsequently resurvey all adults in the network villages (ie, 2–3 months after the first network village survey round).

### Study participants

Groups included in this research comprise adults (aged 18 years and above) in rural Salavan and rural Chiang Rai. We focus on Thailand and Lao PDR because they are situated in a region that experiences high rates of antibiotic use and increasing resistance.^4 33 34^ Compared with Lao PDR, Thailand exhibits a more advanced economic and health system context and more established AMR stewardship. A comparative study of these LMIC contexts (Lao PDR and Thailand) therefore offers interesting lessons for domestic and global antibiotic policy. We focus on adults because they account for much of the popular antibiotic demand and typically acquire and administer antibiotics on behalf of children.^35^ Lastly, we focus on rural areas of Chiang Rai and Salavan because their formal and informal health systems face relatively high infrastructural, human resource, financial and regulatory constraints; while their inhabitants are more often characterised by economic, social and spatial marginalisation. This does not automatically mean that our entire study population qualifies as ‘marginalised’ since we define marginalisation in relative terms of wealth (eg, bottom quintiles of household asset and amenity indices), social position (eg, within village social networks) and geography (eg, distance to urban centres). However, it is important to note that our study implications will speak to rural areas with their specific constraints and patterns of marginalisation, which are systematically different from urban settings.

### Data collection

**Figure 2:**
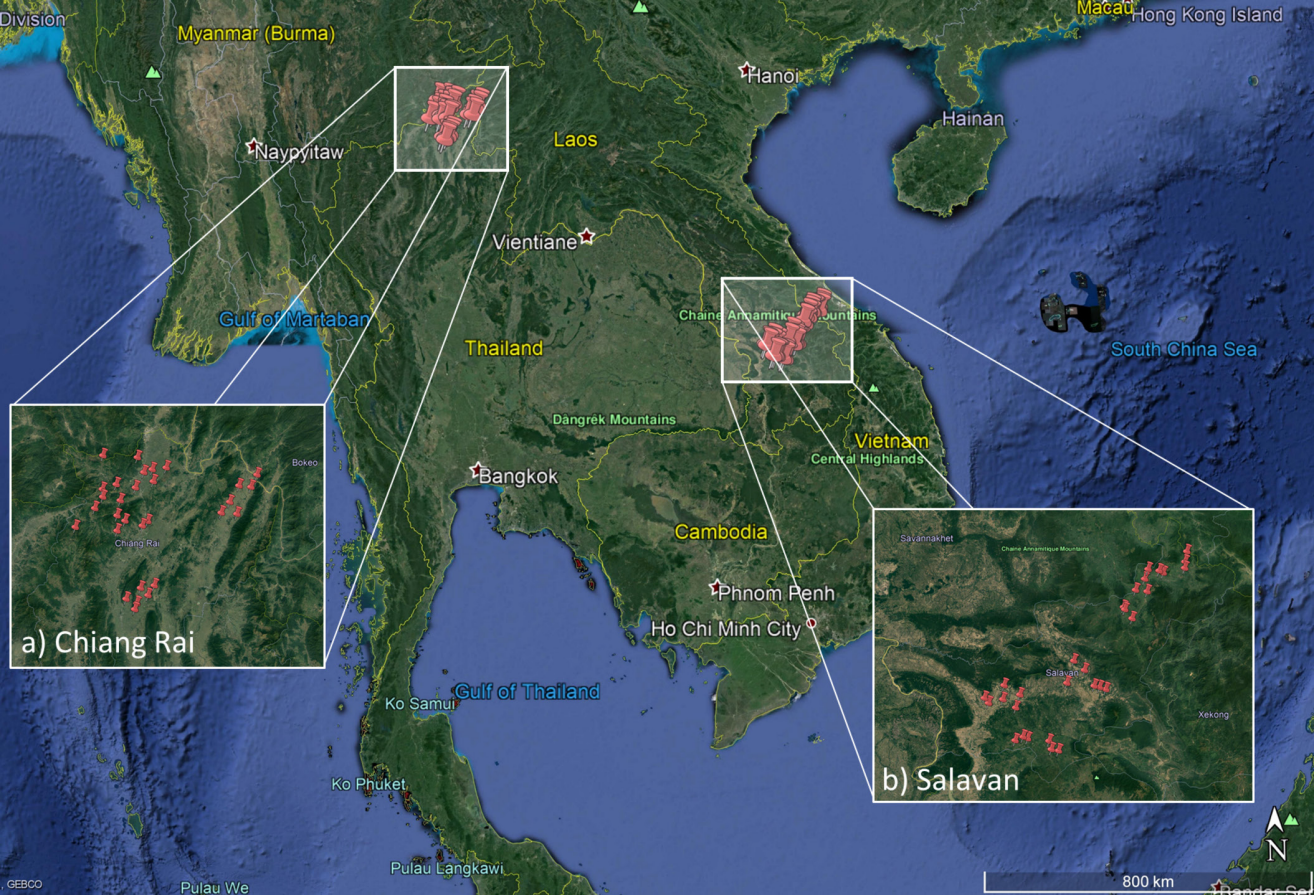
Field sites and sampled villages in Thailand and Lao PDR.

The district-level representative survey will be conducted in a three-stage stratified cluster random sampling design. A cluster sample is necessary to ensure the logistical and financial feasibility of the survey, and we aim to reduce its negative implications for the effective sample size through stratification, which helps to increase the information contained in each cluster.^36^ The first stage involves the random selection of 30 villages (clusters) across five purposively selected districts in each site, stratified by their distance to the nearest urban centre (using data from National Geospatial Intelligence Agency^37^). Figure 2 depicts the resulting village samples (source: authors, adapted from Google^38^ 2017 map data from Landsat/Copernicus). The second stage enumerates all residential buildings within the selected villages using satellite imagery from *Google Maps* and *Bing Maps*, of which we sample 5% of the buildings (but at least 30 houses) in a stratified interval sampling approach to ensure spatial representativeness. During the survey implementation, the third sampling stage will select randomly one respondent for every five adults in each chosen house.

The sampling strategy for the community-level social network census surveys involves the purposive selection of three comparable villages in both countries. Selected in consultation with local stakeholders, guiding criteria for selection were (1) village size and structure, (2) remoteness and road accessibility, (3) economic status as approximated by village-level infrastructure and facilities, (4) ethnic composition and (5) number and location of health facilities within a 2 km radius. The villages are estimated to have a size of 100–200 households with 2.9 adults per rural household in Lao PDR (ranging from 1.8 to 4.6 per village) and 2.4 in Thailand on average (ranging from 1.6 to 4.3 per village).^39 40^ Within the selected communities, all households will be approached, their adult members enumerated and invited to participate.

**Figure 3:**
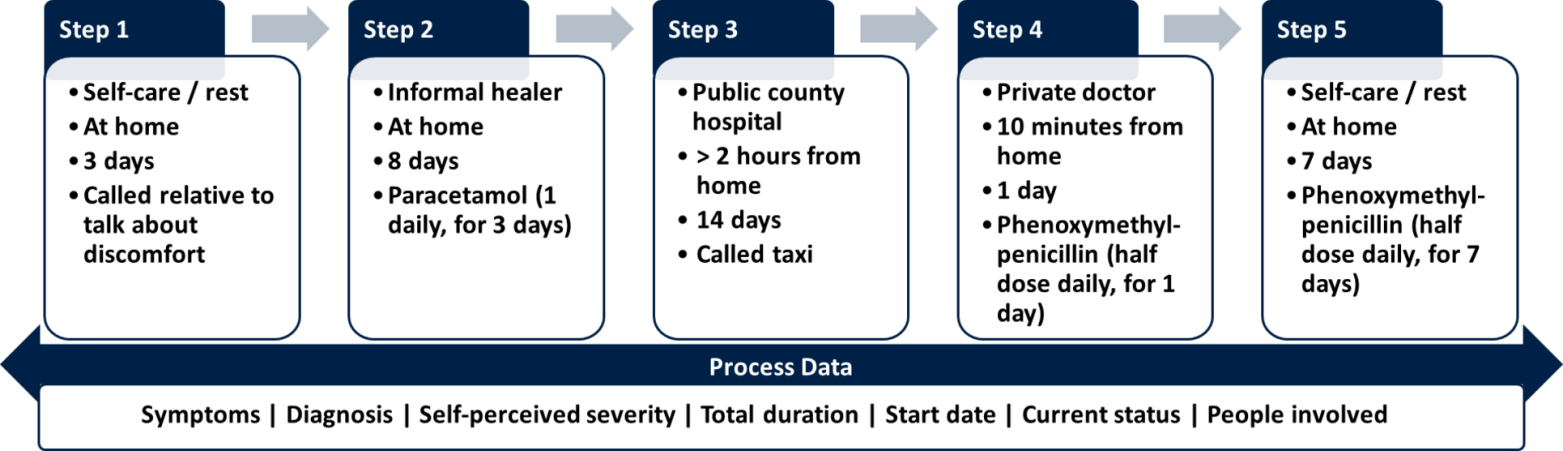
Example of treatment-seeking pathway data collected in the survey.

Our survey instrument will be a 45 min questionnaire that captures people’s complex healthcare-seeking pathways and their medicine use therein. An important feature of this instrument is the collection of self-reported sequential healthcare pathway data for acute illnesses and accidents that occurred in the 2 months prior to the interview. As shown in figure 3 (source: authors, adapted from Haenssgen and Ariana^41^), we will subdivide an illness into discrete steps of activities and record their type, duration and location; with whom the patient interacted during the healthcare activity; whether the patient used any medicines during the step (elicited using a ‘drug card’ containing the most common local medicines), their source, and how long, often, and at what dosage they were taken and whether, why, and by whom any kind of health-related mobile phone, Internet, media or vehicle use took place.iii We also collect data on the social and economic background of the respondents as well as information about people’s existing conceptions of and attitudes towards antibiotics. In the case of the social network censuses, we will ask additional questions to construct four different kinds of (health-related) social networks:

- (Health) communication networks: people within and outside the village with whom the respondent interacts and talks about health (elicited in first network survey round).
- Incidence network: places where the respondent typically interacts with other villagers (elicited in first network survey round).
- Help network: contacts who are activated during an illness (elicited in both network survey rounds).
- Information network: people with whom the respondent talked about the public engagement activity (elicited in second network survey round).

The questionnaire will be administered through tablet-based electronic data collection by locally recruited survey teams comprising six enumerators and two survey supervisors per country. The survey period will be between November 2017 and April 2018, which is the post-rice-harvest dry season in both field sites. This season was chosen for village accessibility (landslides and floods are common during the rainy season) and the availability of villagers for interviews (villagers often reside temporarily near their rice fields during the planting and harvest seasons). Due to the temporal focus on one season, our survey is therefore not able to capture seasonal change affecting the epidemiological environment, internal migration, healthcare-seeking patterns and interactions within social networks.

We will pilot the questionnaire to identify respondents’ understanding of the survey questions and the range of possible answers. Between 10 and 30 1 hour cognitive interviews per site will support this process and enable insights into how respondents understand survey questions and how they arrive at their answers.^42^ The qualitative data generated through the cognitive interviews will also facilitate the interpretation of the quantitative survey results.

Two further sources of data will complement our survey data. First, in order to understand antibiotic-seeking behaviour in the local health systems, separate checklists will help us to gather observational information about the location of formal and informal healthcare providers in each village. Second, we will estimate patient load and peak demand for public healthcare services by accessing secondary administrative data from public primary care facilities that cater to the sampled villages.

### Analysis

The data analysis techniques to inform our research questions include:

- RQ1: sequence analysis to describe and understand linear series of events.^41 43 44^
- RQ1–3: multilevel regression analysis to test the relationship between antibiotic use as a dependent variable and a range of established determinants of treatment-seeking behaviour as independent variables.^45^
- RQ2: social network analysis (network-based event history and relational event sequence analysis) to examine how behaviours and beliefs across a social network relate to individual behaviours and beliefs and how this relationship persists over time.^46–48^
- RQ3: latent class analysis to identify (1) common symptoms associated with problematic antibiotic usage, (2) the characteristics of populations who are likely to exhibit problematic antibiotic behaviours and (3) contextual conditions predicting adverse behaviours, all of which may help guide future interventions and policies.^49^

Related in particular to Research Questions 1 and 2, we will further examine six hypotheses about antibiotic use among the general population.

H1. Marginalised groups have fewer means to access formal treatment, which increases their likelihood to rely on over-the-counter medicines including antibiotics as an alternative solution.

H2. Technology use increases access to formal healthcare providers but is directed towards those who are more inclined to prescribe antibiotics.^iv^

H3. Awareness about ‘rational antibiotic use’ alone has only a minor influence on antibiotic usage behaviour if patients are economically, socially or spatially marginalised.

H4. In the absence of competing healthcare practices, new antibiotic-related behaviours diffuse through social networks.

H5. Pre-existing competing practices subdue the spread of new antibiotic-related behaviours within the community network if no ‘critical mass’ can be achieved.

H6. Peak demand for scarce high-quality healthcare providers drives less competitive (ie, more marginalised) patients into behaviours that are more likely to involve adverse antibiotic use.

Note that these hypotheses do not intend to declare the behaviour of marginalised groups to be ‘irrational’. Rather, we hypothesise that the behaviour of marginalised groups is subject to greater healthcare access constraints, owing to which antibiotic use might be more likely. Whether this is indeed the case and whether these behaviours are less appropriate than otherwise are empirical questions that we hope to inform through our survey.

## Discussion

### Ethical considerations

#### Informed consent

We received a waiver for written consent requirements in order to not unfairly exclude illiterate population subgroups in our field sites^50^ and to ensure trust between the researcher and the rural respondents.^51^ Instead of participant-dated signature, we follow a verbal consent process in which (1) we seek permission from village leaders to carry out our survey in their villages; (2) the survey fieldworker reads out (and records on audio tape) an oral consent script to the potential respondent and provides them with a printed copy of the participant information sheet; (3) the survey fieldworker asks the respondent to state her or his consent, name and date on audio record and (4) the survey fieldworker personally signs and dates a written record of oral consent. We provide a detailed justification and explanation of this verbal consent process in online Supplementary appendix 1.

10.1136/bmjgh-2017-000621.supp1 Supplementary file 1

#### Privacy and confidentiality

Further ethical considerations in this study relate to privacy and confidentiality. The data collected in this study include self-reported health and economic information. Personal contact details will be stored separately from the data sets in order to match data from repeated network survey rounds. Any identifying information will be deleted from the analytical data set or coded into anonymous respondent numbers for the social network census survey data set. Geographical data allowing household identification will be translated into distance measures and a village-centred metric coordinate system (similar to the Universal Transverse Mercator system). Should village layouts prove idiosyncratic so that the metric coordinate system enables identification, we will withdraw these data from the data sets.

### Proposed impact

The academic impact of our study pertains to antibiotic-related behaviour and its relationship to marginalisation, technology and social relationships. Our innovations therein are theoretical (development of the activity space framework to conceptualise and situate people’s antibiotic access and use during illness), methodological (sequence analysis for healthcare pathways) and empirical (novel insights into the impact of marginalisation, technology and knowledge on antibiotic usage). In addition, we will build capacity for social research in AMR, for instance, through four internships for local candidates from Southeast Asia who consider interdisciplinary academic careers, and our project aims to influence the global health discourse about AMR, for example, by hosting four 8-week student placements with the MSc International Health and Tropical Medicine (IHTM; a global health degree at the University of Oxford).

In conclusion, social research in AMR is nascent, but our unprecedentedly detailed data on microlevel treatment-seeking behaviour promises to contribute to understanding behaviour beyond awareness and free choice, highlighting, for example, decision-making constraints, problems of marginalisation and lacking access to healthcare and competing ideas about desirable behaviour.

## Footnotes

i Information is also unlikely to remain static during the diffusion process; utilisation and (re-)interpretation of educational messages can alter nature, meaning and value of this information over time and across people. Our focus on healthcare-seeking behaviour and attitudes towards antibiotics can capture unintended behavioural responses to a limited extent, but our survey is unable to study the implications and mechanisms underlying such potential transformations comprehensively (for which qualitative and ideally ethnographic research would be better suited). We thank an anonymous reviewer for pointing this out.

ii While we do not expect regular social networks to evolve as a result of our educational activity, we will be able to ascertain whether people activate different social contacts when seeking treatment before and after our activity. In addition, we will also learn whether respondents who did not participate in the activity learnt about it through their regular social network or from other contacts.

iii Due to language ambiguities in the term ‘antibiotic,’ we do not ask people directly whether they took an antibiotic, but rather which medicine they received and used at each step in the treatment-seeking process. Pilot interviews suggested that a part of the rural population is able to describe medicine unambiguously (eg, using colloquial or technical terms for antibiotics), while other groups do not know what medicine they received. In the latter case, we triangulate their responses with their descriptions of antibiotics (eg, as ‘sore throat medicine’, which we also capture in the questionnaire), and we ask for descriptions of these medicines (eg, ‘a shot’, ‘a green-blue capsule’). We then code the medicines for sensitivity analysis into categories ‘likely to be an antibiotic’, ‘unlikely to be an antibiotic’ and ‘cannot ascertain type of medicine’ according to common medicines used in the field sites.

iv The interest in technology use is rooted in our activity space framework, according to which different healthcare providers (eg, private doctors) are more responsive to patients’ use of, for example, mobile phones, which can affect treatment-seeking pathways and, potentially, antibiotic use.
