# Supplementary material for: Antibiotics and activity spaces: protocol of an exploratory study of behaviour, marginalisation and knowledge diffusion
Source: BMJ Glob Health. 2018 Mar 28;3(2):e000621. doi: 10.1136/bmjgh-2017-000621 (PMC5884330; doi:10.1136/bmjgh-2017-000621)
Supplement: Supplementary file 1 [file bmjgh-2017-000621supp001.pdf]

Research Protocol: Antibiotics and Activity Spaces: An Exploratory Study of Behaviour, Marginalisation, and Knowledge Diffusion

APPENDIX 1: JUSTIFICATION FOR VERBAL CONSENT

We received a waiver for written consent requirements in order to not unfairly exclude illiterate population sub-groups in our field sites [1], and to ensure trust between the researcher and the rural respondents [2]. Instead of participant-dated signature, we follow a verbal consent process: (1) we seek permission from village leaders to carry out our survey in their villages, (2) the survey fieldworker reads out (and records on audio tape) an oral consent script to selected survey respondents and also provides them with a printed copy of the Participant Information Sheet, (3) the survey fieldworker asks the respondent to state her or his consent, name, and date on audio record, and (4) the survey fieldworker personally signs and dates a written record of oral consent. This proposed process:

(a) responds to the nature of this study, which takes place in varied populations where 13% of adults in rural Northern Thailand and 27% in rural Salavan do not have any formal education and can be assumed to be illiterate [3 4].

(b) appreciates that signatures and thumb prints in our rural field sites can over-formalise the relationship between the researcher and the respondents, which can make respondents uncomfortable and discourage participation in the research.

(c) complies with International Ethical Guidelines for Biomedical Research Involving Human Subjects for minimal risk research among partly illiterate populations in non-Western research contexts [5].

(d) ensures that respondents still have the opportunity to give informed consent freely, while enabling the transparent monitoring of the consent process through audio records and signed records of oral consent.

Our approach is not unique; it is based on existing guidelines and practice in biomedical and social research in low- and middle-income countries. For example, in a survey of 203 researchers from 90 countries across low- and middle-income Asia, Africa, and South America, Hyder and Wali [6] report that one-third of the researchers use verbal consent processes. According to their analysis, verbal consent was more often employed in research with low-literacy populations, by non-physician researchers, and in lower-risk behavioural and cultural research. A survey among 387 US researchers working in low- and middle-income countries yielded a comparable fraction of 41% who obtain verbal consent in their studies [1]. Examples from empirical bioethics research demonstrate that verbal consent can be a viable alternative to written consent in low-risk health-related research with ethnically and culturally diverse groups:

- Killawi, et al. [7] carried out a qualitative research study in Qatar on health services quality among the general population. The authors obtained audio-recorded verbal consent (together with providing a written participant information sheet) for cultural reasons: "Signatures are usually reserved for formal transactions associated with major life events" [7].
- Lloyd, et al. [8] obtained verbal consent for a qualitative study among ethnic minority diabetes patients in the UK. Respondents were given an information sheet together with audio-recorded information about the research. They were then asked if they had any questions, the consent-taking process was explained, the respondents were asked to repeat the consent form items as read out by the researcher, and the respondents would then state their name, time, and date to give their consent. The process was documented via audio recording and participants would retain a copy of the audio record. Based on interviews with the participants, the authors concluded that, "this method of obtaining consent was found to be acceptable to all those [respondents] taking part" [8].
- Tindana, et al. [9] carried out qualitative research in Ghana about informed consent processes, indicating how illiterate respondents preferred verbal consent because "the paper

> will not mean anything to me." However, the study also highlighted that written documents can serve as a reminder for respondents that they participated in the study, and that community leaders play an important role as gatekeepers in rural research.

More generally, researchers and research ethics guidelines agree that verbal consent can be an alternative to written consent where (a) the research carries only minimal risks, (b) literacy and the cultural context make signatures and thumb prints problematic for equal participation in research, and (c) the consent process is documented adequately [1 2 10-12]. For example, the Nuffield Council on Bioethics [13] and the National Bioethics Advisory Commission [14] appreciate that signing documents can over-formalise the relationship between the researcher and the participant, thereby undermining trust and discouraging participation. The same concerns can apply when using thumb prints to document consent [15 16]. At the same time, Marshall [2] maintains that, "in many cultural settings, agreements based upon trust do not require a signature." Altogether, these reports suggest that alternative arrangements to written consent are possible but require "safeguards" like an audio recording of the respondent's consent, or a signed statement by the researcher that the study had been explained and that the participant had given her or his consent. Bhutta [12], who reviewed various research ethics guidelines, seconds this argument and states that, "Using the improved technology of video and audio recordings during the consent process may carry the dual benefits of documenting consent as well as acting as a mechanism for oversight by ethics review committees."

In light of the varying levels of literacy in our rural field sites, the risk of alienating participants who are only very infrequently requested to sign documents, and the low-risk nature of our social survey, we substituted the written consent process with audio-recorded and documented verbal consent. Following permission of village leaders to conduct our survey, we approach the sampled households and select the respondents accordingly. For the selected respondents, the fieldworker reads an oral consent script, containing the same information as the Participant Information Sheet (the respondent receives a printed copy). The script and PIS are available in Thai and Lao and include information on

the exact nature of the study; what it will involve for the participant; the implications and constraints of the protocol; data sharing arrangements; and the risks and benefits involved in taking part. The fieldworker thereby explains that the participant is free to withdraw from the study at any time for any reason without prejudice to future care, and with no obligation to give any reason for withdrawal. The reading of the script is audio recorded in order to document the consent process.

The participants are allowed as much time as they wish to consider the information, and they have the opportunity to question the field investigator to decide whether they will participate in the study. Verbal Informed Consent is then obtained by the respondent confirming that they wish to take part in the study, stating their name and confirming whether the project has been explained to them, whether they have received the information sheet, whether they give the interviewer the permission to survey them, and whether they agree with the study’s data sharing arrangements. The survey fieldworker then stops the recording and completes and signs the record of oral consent.

REFERENCES

1. Dawson L, Kass NE. Views of US researchers about informed consent in international collaborative research. Social science & medicine 2005;**61**(6):1211-22 doi: http://doi.org/10.1016/j.socscimed.2005.02.004.

2. Marshall PA. Informed consent in international health research. Journal of Empirical Research on Human Research Ethics 2006;**1**(1):25-41 doi: 10.1525/jer.2006.1.1.25.

3. UNICEF. Thailand: multiple indicator cluster survey 2012. Bangkok: UNICEF, 2013.

4. UNICEF. Lao PDR: Lao social indicator survey (LSIS) 2011-2012 (multiple indicator cluster survey / demographic and health survey). Vientiane: UNICEF, 2012.

5. CIOMS. International ethical guidelines for biomedical research involving human subjects. Geneva: Council for International Organizations of Medical Sciences, 2002.

6. Hyder AA, Wali SA. Informed consent and collaborative research: perspectives from the developing world. Developing World Bioethics 2006;**6**(1):33-40 doi: 10.1111/j.1471-8847.2006.00134.x.

7. Killawi A, Khidir A, Elnashar M, et al. Procedures of recruiting, obtaining informed consent, and compensating research participants in Qatar: findings from a qualitative investigation. BMC Medical Ethics 2014;**15**(9) doi: 10.1186/1472-6939-15-9.

8. Lloyd CE, Johnson MR, Mughal S, et al. Securing recruitment and obtaining informed consent in minority ethnic groups in the UK. BMC Health Services Research 2008;**8**(68) doi: 10.1186/1472-6963-8-68.

9. Tindana PO, Kass N, Akweongo P. The informed consent process in a rural African setting: a case study of the Kassena-Nankana district of northern Ghana. IRB 2006;**28**(3).

10. Alaei M, Pourshams A, Altaha N, et al. Obtaining informed consent in an illiterate population. Middle East Journal of Digestive Diseases 2013;**5**(1):37-40.

11. Tekola F, Bull SJ, Farsides B, et al. Tailoring consent to context: designing an appropriate consent process for a biomedical study in a low income setting. PLOS Neglected Tropical Diseases 2009;**3**(7):e482 doi: 10.1371/journal.pntd.0000482.

12. Bhutta ZA. Beyond informed consent. Bulletin of the World Health Organization 2004;**82**(10):771-77.

13. Nuffield Council on Bioethics. The ethics of research related to healthcare in developing countries. London: Nuffield Council on Bioethics, 2002.

14. National Bioethics Advisory Commission. *Ethical and policy issues in international research: clinical trials in developing countries*. Bethesda, MA: National Bioethics Advisory Commission, 2001.

15. Sugarman J, Popkin B, Fortney J, et al. International perspectives on protecting human research subjects. In: National Bioethics Advisory Commission, ed. Ethical and policy issues in international research: clinical trials in developing countries. Bethesda, MA: National Bioethics Advisory Commission, 2001:E-1-E-30.

16. Upvall M, Hashwani S. Negotiating the informed-consent process in developing countries: a comparison of Swaziland and Pakistan. International Nursing Review 2001;**48**(3):188-92 doi: 10.1046/j.1466-7657.2001.00063.x.
